# Neuroscientific Methods for Exploring User Perceptions While Dealing With Mobile Advertising: A Novel and Integrated Approach

**DOI:** 10.3389/fnrgo.2022.835648

**Published:** 2022-04-08

**Authors:** Marco Mancini, Patrizia Cherubino, Gianluca di Flumeri, Giulia Cartocci, Ana Martinez, Alessandro Sanchez, Chiara Santillo, Enrica Modica, Alessia Vozzi, Vincenzo Ronca, Arianna Trettel, Gianluca Borghini, Fabio Babiloni

**Affiliations:** ^1^BrainSigns, Rome, Italy; ^2^University of the International Studies of Rome (UNINT), Rome, Italy; ^3^Department of Molecular Medicine, Sapienza University of Rome, Rome, Italy; ^4^Department of Communication and Social Research, Sapienza University of Rome, Rome, Italy; ^5^Department of Anatomical, Histological, Forensic and Orthopedic Sciences, Sapienza University of Rome, Rome, Italy; ^6^College of Computer Science and Technology, Hangzhou Dianzi University, Hangzhou, China

**Keywords:** EEG, eye tracking, autonomic recording, online users, mobile advertising, display advertising, native advertising

## Abstract

Display and native ads represent two of the most widely used digital advertising formats employed by advertisers that aim to grab the attention of online users. In recent years, the native format has become very popular because it relies on deceptive features that make harder the recognition of its advertising nature, reducing avoiding behaviors such as the banner blindness phenomena, traditionally associated to display advertising, and so increasing its advertising effectiveness. The present study, based on a forefront research protocol specifically designed for the advertising research on smartphone devices, aims to investigate through neurophysiological and self-reported measures, the perception of display and native ads placed within article webpages, and to assess the efficacy of an integrated approach. Eye-tracking results showed higher visual attention and longer viewing time associated with native advertisements in comparison to traditional display advertisements, confirming and extending evidence provided by previous research. Despite a significantly higher rate of self-reported advertising intent was detected for articles containing display ads when compared to articles containing native ads, no differences have been found while performing the same comparison for the neurophysiological measures of emotional involvement and approaching motivation of for the self-reported measures of pleasantness and annoyance. Such findings along with the employment of an innovative research protocol, contribute to providing further cues to the current debate related to the effectiveness of two of the most widely used digital advertising formats.

## Introduction

In the last decade, we have witnessed a significant shift in the advertising world from traditional media to online media, leading to a huge growth in digital advertising expenditure worldwide. For the first time, in 2015, in the UK, digital media took a 50% share of advertising spending (eMarketer, 2015), while in 2016, in the US, digital revenues surpassed those of cable and broadcast television (IAB, 2016). Another significant change was represented by the huge growth of mobile revenues at the expense of the desktop revenues, this latter being dominant till 2016, when digital revenues coming from mobile devices exceeded desktop ones, making in 2018 (full year) up 65.1% of total internet advertising revenues (Silverman, 2019). Currently, we are living in the “Mobile Age,” a world with more than 3.2 billion smartphone users (Statista, 2018) that, thanks to the internet access, could be potential viewers of digital advertisements at any time. In this context, the improved ability to successfully match advertisers and consumers allowed by multisided platforms, detailed data, and algorithms led to a substantial transformation of the advertising business programmatic advertising that allows to collect consumers data, monitor ads impressions, and automate the buying and selling of ads in order to reach an effective target audience (Wojdynski, 2016a; Bounie et al., 2017); it needs to deal with two main issues: the banner blindness phenomenon and the current difficulties in measuring affective and cognitive processes elicited by the ads.

One of the historical metrics widely used to evaluate digital advertising campaigns is the Ad impression, which has been meticulously refined in recent years to achieve more precise measurements, combining many pre-established criteria, such as the percentage of pixels within the viewable space and the length of time the ad is in the viewable space. In 2014, the Interactive Advertising Bureau (IAB) introduced the concept of viewable impression, different from the previous definition of impression (or served impression; MediaRatingCouncil IAB, 2014): from 2014, an ad impression was considered viewable only if the ad was contained in the viewable space of the browser window. Later on, in 2016, the IAB released another guideline specifically about the viewability of an Ad on mobile devices (MRC, 2016) stating that a mobile impression was viewable if the ad was contained in the viewable space of the device, either within an in-focus web browser (Web View) or a fully downloaded, opened, initialized application. In particular, nowadays, a viewable mobile ad needs to meet the following requirements (Interactive Advertising Bureau Technology Laboratory Mobile Marketing Association Media Rating Council, 2017):

- Pixel Requirement: an amount greater than or equal to 50% of the pixels in the advertisement has to be located on an in-focus browser or a fully downloaded, opened, initialized application, on the viewable space of the device.
- Time Requirement: The time the pixel requirement is met has to be greater than or equal to one continuous second, post ad render.
- Valid Traffic: viewable impressions will be counted only in case of valid traffic, excluding the traffic generated by bots and other automated tools.

Despite the promising development of the above-mentioned requirements, half of the ads purchased by the advertisers are never seen by internet users, leading to a huge budget waste (Bounie et al., 2017). One of the reasons ads may fail to achieve their goal is the so-called banner blindness phenomenon, primarily related to banner ads and that became known 4 years later the first commercial banner appeared on a website (Wired website in 1994; Ausiello, 2015). The term “banner blindness,” coined by Benway (1998), refers to the user's tendency to ignore banner-like information or anything else that resembles an advertisement. Such phenomenon relies on the increasing abilities of users in identifying banner ads and their persuasive purposes, along with the user development of strategies to resist them (Fransen et al., 2015) or avoid them entirely (Drèze and Hussherr, 2003; Cho and Cheon, 2004; Lee and Ahn, 2012). In a series of eye-tracking studies (Nielsen, 2007; Pernice, 2018), the Nielsen Norman Group stated that the banner blindness phenomenon is a type of selective attention and that users who navigate websites have learned how to pay attention to elements that can be helpful and how to ignore the one's void of information (Pernice, 2018). To increase the user attention toward the ad and deal with the banner blindness phenomena, strategies such as animation can be adopted (Griffith et al., 2001), although it could lead to an increase in the cognitive effort required for processing the ad and negatively affect memory performance associated to the ad recall (Bayles, 2002; Hong et al., 2004; Burke et al., 2005), along with a wide variety of paid advertising formats, more difficult to identify by the user (Mitra et al., 2008; Campbell et al., 2014). Regarding the introduction of new digital advertising formats, the so-called “native” format matches the look, feel, and function of the media format in which it appears (Wojdynski, 2016a; IAB, 2019) and such “deception” makes it more difficult to recognize by users (Most et al., 2001; Wasserman, 2013; Anni, 2014; IAB, 2019). The use of ambiguous words, small in size, like “sponsored” or “branded” content, represents the only cue (AdAge, 2014) for the user to distinguish between the advertisement and the native feed of the site, and for this reason, some researchers were wondering if the native format actually leads to an increased engagement or to an increased deception (Wojdynski, 2016a). The success of native advertising was confirmed by an eye-tracking study conducted by Sharethrough/IPG Media in 2015, showing that consumers looked at native ads 53% more frequently than display ads. Furthermore, native ads registered 18% higher lift in purchase intent and 9% lift for brand affinity responses than banner ads and 32% of consumers were willing to share a native ad with their family or friend, while only 19% would have shared a banner ad (Sharethrough, 2015). Beyond the success of native advertising, some authors warn that deceptive practices can lead consumer to avoid, distrust, or negatively perceive ads (Fransen et al., 2015; Wojdynski, 2016a; Taylor, 2017; Han et al., 2018; Iversen and Knudsen, 2019), negatively affecting the perceptions of the article, of the publisher, and of the advertising institution, and decreasing the intent to share the article (Wojdynski, 2016b; Amazeen and Wojdynski, 2020). In addition, a study reported that consumers actually evaluate banners more positively than article-style native ads, in terms of attitude and credibility (Harms et al., 2019).

A total of 40 years have passed since Zajonc disclosed the fundamental role of emotion in advertising (Zajonc, 1980), paving the way to studies that would have described emotion as an important mediator of cognitive and behavioral consumer responses when processing advertising (Batra and Ray, 1986). If it is clear that accurate measurement of emotions would be essential (Poels and Dewitte, 2011), the most widely used techniques in this context are represented by the traditional self-reports (Velásquez et al., 2011; Li et al., 2018), very convenient to use, but mainly relying on how users describe advertisements (Edell and Burke, 1987), sensible to biases (Paulhus, 2002), and hardly capable to measure affective and cognitive processes elicited by ads (Edell and Burke, 1987; Mano, 1996; Cherubino et al., 2019). In recent years, the exponential production of scientific articles related to the neuromarketing field generated new knowledge related to the application of neuroscientific tools in advertising testing (Cherubino et al., 2019) that may fill this gap and may help provide a better understanding of processes such as attention and emotion that play a crucial role in evaluating the effectiveness of ads (Edell and Burke, 1987; Harrington et al., 2006; Milosavljevic and Cerf, 2008). Even if neuroscientific technologies have been applied since the 1960's in consumer research (Cherubino et al., 2019), the current technological advances, at the basis of modern neuroscience, allow to deeply explore emotions and cognitive processes while users are interacting with a stimulus, like a website or an app by desktop or mobile devices. A recent neuroscientific study (Neurons Inc, 2021) involved the recordings of the eye-tracking and the electroencephalography (EEG) of 1,049 participants and turning in the world's largest consumer neuroscience study on mobile devices, providing a better understanding of the impact of digital advertising in terms of emotion and attention. This study revealed that 25% of ads are seen on mobile screens vs. the 5% on desktops, that the advertisement starts to generate emotional and cognitive responses only after 700 ms, and that 50% of the ads produce an emotional response if the ad is seen for at least 1,000 ms, leading to the definition of the “1-s ad strategy.” The integration of techniques such as the eye tracking and the EEG can provide real-time information to detect where a person is looking at (Vila and Gomez, 2016), which sequential strategies are being used (Goldberg and Helfman, 2011) and to identify approaching vs. avoiding behaviors toward a stimulus of interest such as a digital ad (Davidson, 1995; Davidson and Hugdahl, 1996; Di Flumeri et al., 2018; Modica et al., 2018).

Our study aims to demonstrate the potentiality of a forefront experimental protocol that involves the integration of neuroscientific tools and traditional techniques, focusing on the comparison of display and native advertising format in terms of visual attention, emotional involvement, and approaching behavior, while browsing several web pages by smartphone. Considering that only a few studies have previously addressed similar topics by the use of an integrated set of neuroscientific and standard tools (Sharethrough, 2015; Neurons Inc, 2021) and that previous research has also shown some counter-current results highlighting a more positive evaluation of display ads vs. native ads (Harms et al., 2019) or raising concerns related to the deceptive features at the base of native ads as a source of negative effects (Fransen et al., 2015; Wojdynski, 2016a; Taylor, 2017; Han et al., 2018; Iversen and Knudsen, 2019), we felt that further investigation was needed. According to our intentions, we developed several web pages showing online articles and containing display or native ads. Our participants, after being instructed, have been asked to read those online articles by smartphone and to fill up a questionnaire at the end of each reading. During the experimental session, the eye tracker has been used to investigate the visual attention and the viewing time associated to native and display ads, based respectively on two of the most widely used eye tracking metrics, namely fixation count (Kukkonen, 2005; Djamasbi et al., 2010; Siyanova-Chanturia et al., 2011; Wang et al., 2014) and visit duration (Dogusoy-Taylan and Cagiltay, 2014; Huddleston et al., 2015; Fashler and Katz, 2016; Katz et al., 2019). The EEG has been used to evaluate cognitive user's behavior during the experience, and in particular to quantify the type of motivation (degree of approach/avoidance) toward articles containing display or native ads, by the use of the Approach Withdrawal (AW) index. Such index, initially developed by Davidson et al. (1990), relies on the hemispheric asymmetries in the alpha frequency range over prefrontal electrodes and represents a widely accepted correlation of approach and withdrawal related motivation (Harmon-Jones et al., 2010; Briesemeister et al., 2013), extensively applied in advertising research (Ohme et al., 2009, 2010; Vecchiato et al., 2010, 2011; Modica et al., 2017; Cartocci et al., 2018, 2019). In addition, heart rate (HR) and galvanic skin response (GSR) have been explored as a measure of the emotional impact generated by articles containing display or native ads, being the HR and the GSR two autonomic parameters sensible to the emotional response (Mauss and Robinson, 2009). The information provided by the GSR and HR have been collected to compute the Emotional Index (EI), a neurophysiological conceptualization of the circumplex model of the effect of Russell and Barrett based on the arousal and valence values (Ariely and Berns, 2010), respectively obtained through the GSR and HR and already applied in advertising research (Vecchiato et al., 2014a,b; Cherubino et al., 2016, 2017).

According to the aforementioned theoretical framework, we aimed to:

1. Verify if native advertising format generates higher visual attention and longer viewing time than display advertising format, considering that previous findings showed a better capability of native ads in reducing visual avoiding behaviors compared to display ads that are highly affected by the banner blindness phenomena (Most et al., 2001; Wasserman, 2013; Anni, 2014; IAB, 2019).
2. Compare articles containing display ads and articles containing native ads in terms of approaching motivation, emotional involvement (EI), pleasantness, annoyance, and advertising intent, considering the concerns raised by some authors related to the deceptive features of native ads as a source of negative effects (Fransen et al., 2015; Wojdynski, 2016a; Taylor, 2017; Han et al., 2018; Iversen and Knudsen, 2019) and some counter-current results highlighting a more positive evaluation of display ads vs. native ads (Harms et al., 2019).
3. Demonstrate the effectiveness of an innovative research protocol specifically designed for advertising research on smartphone devices and based on the integration of both neuroscientific and traditional techniques.

## Materials and Methods

### Participants

The study involved 49 healthy participants (24 females and 25 males; mean age = 26.4 years; SD = 4.9 years). Participants, having no history of mental illnesses or drug abuse, were voluntarily recruited and after the explanation of the study, they signed informed consent, including the authorization related to the use of the video-graphical material (i.e., photos and videos of the experiment). The study was conducted following the principles outlined in the Declaration of Helsinki of 1975, as revised in 2008 and it was approved by the local institutional ethics committee.

### Experimental Protocol

**Figure 1:**
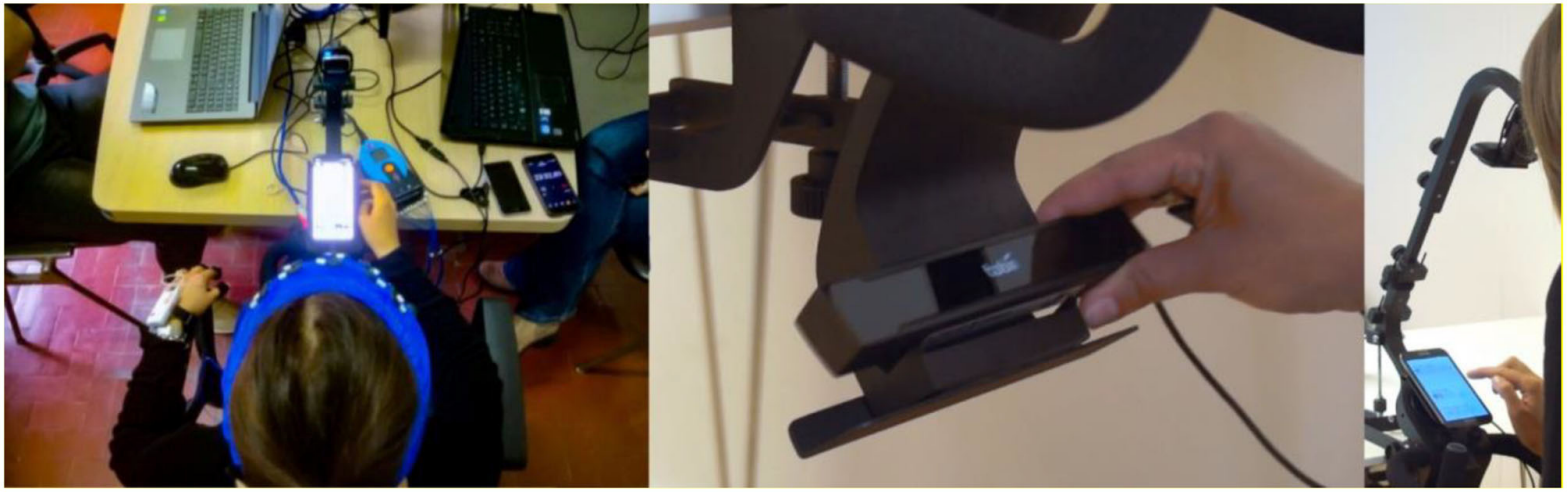
The figure shows on the left side the setup for the electroencephalography (EEG), heat rate (HR), galvanic skin response (GSR), and Eye Tracking recording and on the right side the Mobile Device Stand (Tobii) that was employed specifically for holding the smartphone and performing the eye-tracking recording.

At the beginning of each experimental session, participants were asked to comfortably sit in front of a desk, where a mounting solution designed for facilitating eye tracking on mobile devices [92] and a Samsung Galaxy S8 smartphone (Device Solutions Korea 1, Samsung-ro, Giheung-gu, Yongin-si, Gyeonggi-do, Korea) were present. The recording of the eye movements, brain activity, HR, and GSR have been performed during the entire experimental session (see Figure 1). After 1 min training simply focused on the horizontal and vertical scrolling of the main smartphone's screens to help the participants to familiarized with the specific smartphone model and operative system, the participants were asked to close their eyes for 1 min and then to relax 1 min during the observation of the default Google home page visible on the smartphone screen. This latter recording segment was employed as a baseline.

**Figure 2:**
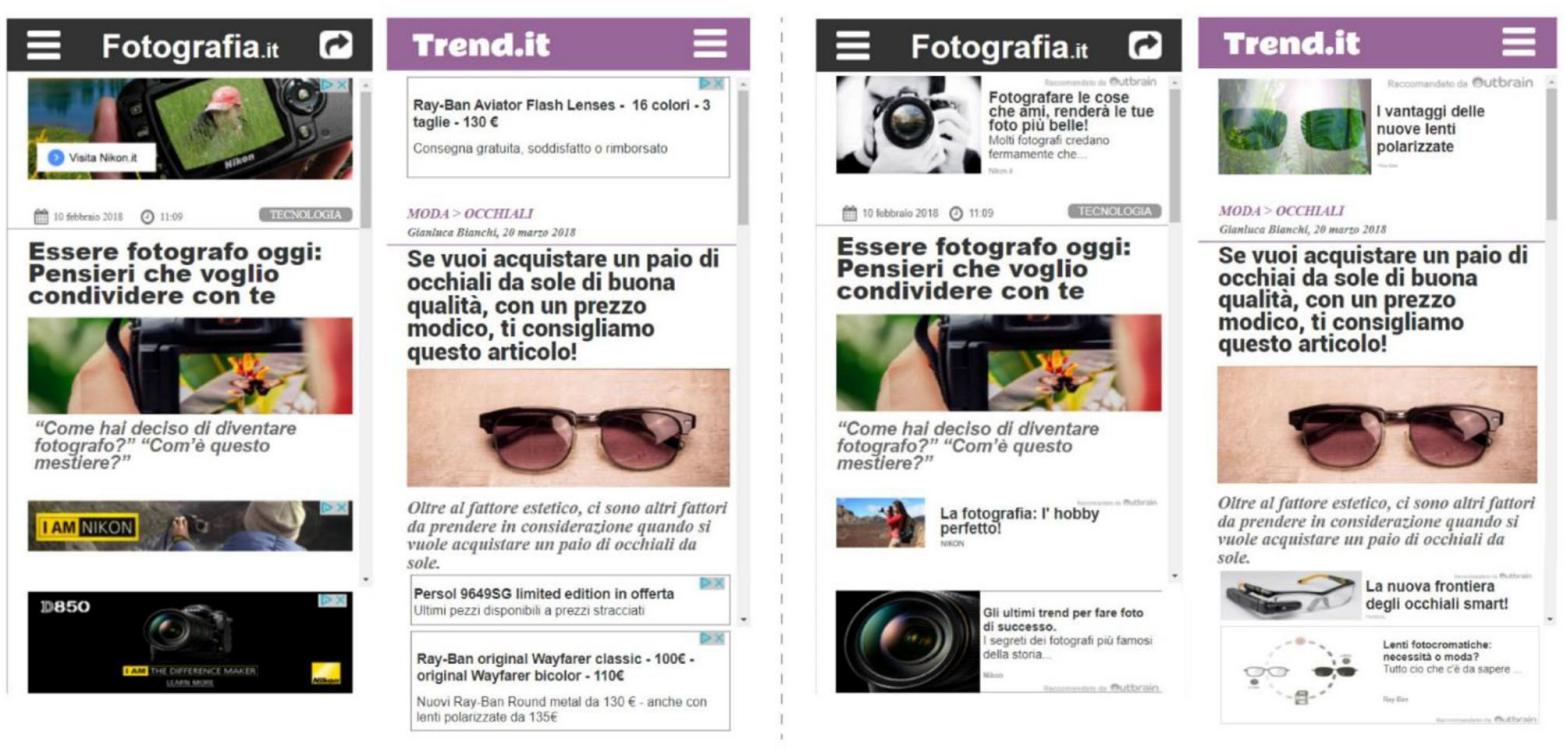
Articles including the ads: on the left side two samples of articles including display ads are shown, while on the right side, the same samples including native ads are shown. Each sample, regardless of the ad format, shows three ads of the total four, since the fourth ad, to be seen by the user, is required to scroll, an action that each participant performed to read the article. The third ad located in each sample was fixed and always visible while scrolling the page.

**Figure 3:**
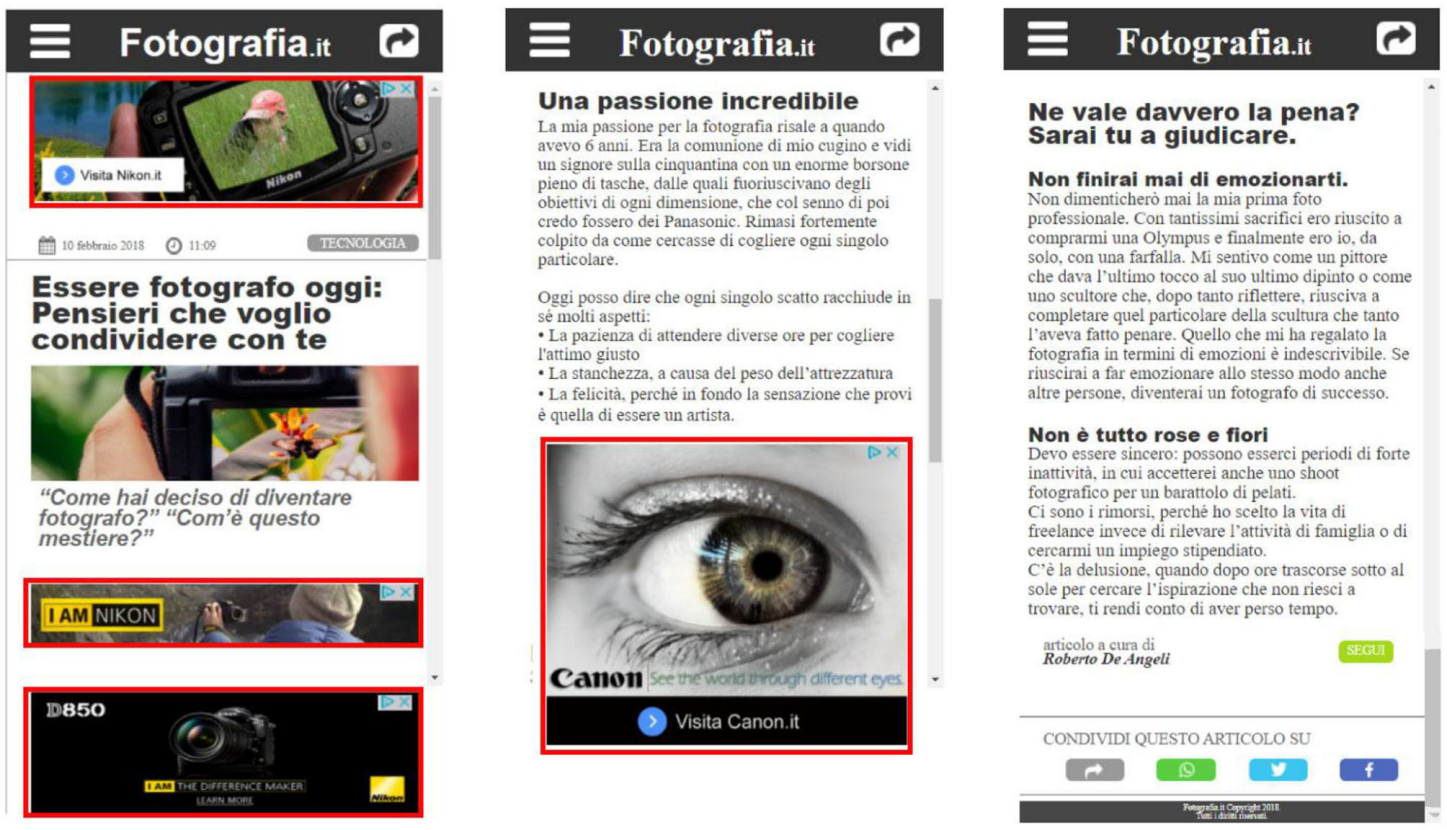
Areas of interest (AOIs)/eye-tracking: the figure shows a sample article where the AOIs, associated with the advertising spaces, are highlighted in red. The third ad, placed in a bottom fixed position, was always visible while scrolling. The AOIs position and dimension were the same for both article formats (display and native).

**Figure 4:**
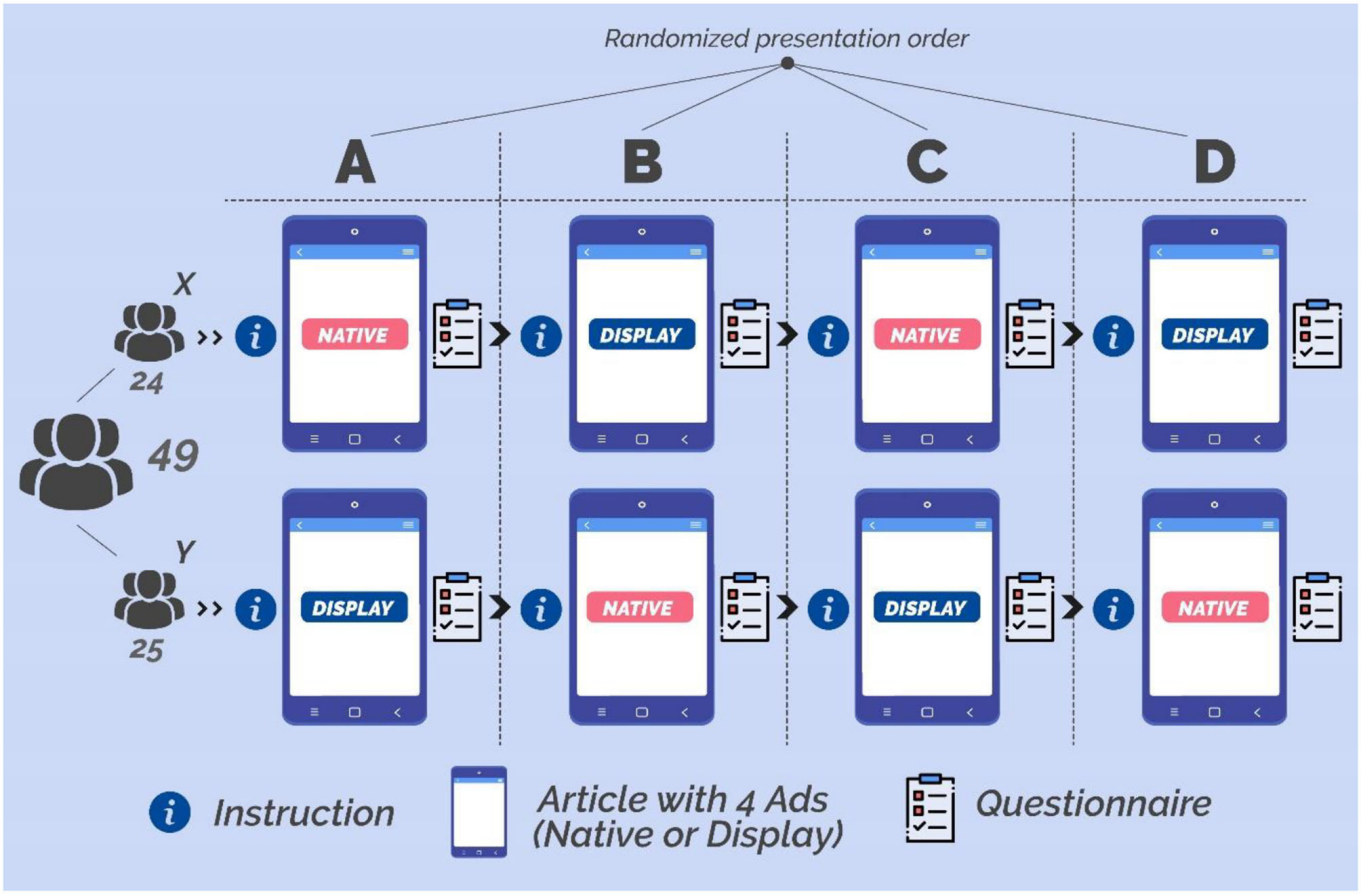
The figure above shows the experimental design. Each participant was invited to read four articles, randomized across subjects, each of them preceded by instruction and followed by a short questionnaire. Regarding the ads, two articles contained four display ads while the other two articles contained four native ads. Each group (“X” ad “Y”) saw the same version of each article except for the advertising format (native/display) that was swapped across groups.

The participants were then invited to visit the “navigation screen,” a landing page we developed to facilitate the navigation throughout different instruction pages and articles containing display or native ads. Each participant was assigned to one of two groups (X and Y) by the use of the random assignment by blocks method, to preserve a similar sample size for both groups (X = 24, Y = 25), generated through a computer-based randomization procedure (“Research Randomizer” tool, available at the following link: https://www.randomizer.org). According to such assignment, each group (“X” and “Y”) saw the same version of each article except for the advertising format (native/display) that was swapped across groups. For instance, the article “A” provided to the group “X” included only native ads, while the same article (“A”) provided to group “Y” included only display ads. Such an approach allowed us to compare the impact of native and display advertisements while controlling all the other article features. In general, each participant was asked to read four articles, where two articles contained four display ads and two articles contained four native ads (see Figure 2 and Figure 3 for more details about the articles containing the ads and related area of interests). The articles faced topics that were consistent with the advertised products, related to sunglasses or cameras. In addition, the order of exposure to each of the four articles was randomized across subjects by the use of the random order assignment method of the “Research Randomizer” tool, to avoid any effect related to the presentation order. At the end of each article reading, participants were asked to rate the pleasantness, the annoyance, and the advertising intent associated with the previous reading, ranging from 1 (minimum) to 7 (maximum; see the whole experimental design in Figure 4). The entire experimental session lasted around 15 min.

### EEG Recording and Signal Processing

Regarding the recording of brain activity, a portable 16-channels EEG system (BEmicro and Galileo software, EBneuro, Italy) was employed. The EEG signal was recorded with a sampling rate of 256 Hz through a frontal headband including 10 Ag/AgCl electrodes placed according to the 10–20 International System (Fpz, Fp1, Fp2, AFz, AF3, AF4, AF5, AF6, AF7, and AF8), all referred to the right earlobe and grounded to the left earlobe. All the skin-electrode impedances were kept below 20 kΩ. The MATLAB software (MathWorks Inc., USA) was employed to process the recorded data offline.

Electroencephalography signals were digitally bandpass filtered by Notch filter (50 Hz), to avoid power line interferences, and a 5th order Butterworth band-pass filter ([2÷30] Hz), to remove high-frequency interferences and the continuous component.

To identify components associated with ocular blinks and saccades artifacts, the independent component analysis (ICA) was applied, in particular employing the SOBI algorithm (Belouchrani et al., 1997). The signal was decomposed in 10 Independent Components (*ICs*, equal to the number of EEG channels), two specific ICs related to eye blinks and saccades artifacts have been visually identified by an expert, and then the EEG signal was reconstructed (Di Flumeri et al., 2016). Afterward, the common average reference (CAR) was applied to re-referenced the EEG signal.

To identify other kinds of artifacts, automatic procedures of the EEGLAB toolbox (Delorme and Makeig, 2004) have been performed on the EEG signal, which was for this purpose segmented into 1-s-long epochs, shifted of 0.5 s to avoid any “boundary effect.” Such automatic procedures have been validated in several previous studies (Aricò et al., 2016, 2017; Di Flumeri et al., 2019) and rely on the following three main criteria:

(i) Threshold criterion: EEG epochs with a signal amplitude exceeding ±100 μV were labeled as an artifact
(ii) Trend criterion: EEG epochs were interpolated to check the slope of the trend within the considered epochs. If the slope of an epoch was higher than 10 μV/s, the considered epoch was marked as “artifact”
(iii) Sample-to-sample difference criterion: The EEG epoch was marked as “artifact” also if the amplitude difference between consecutive EEG samples was higher than 25 μV, inasmuch it would represent a non-physiological variation.

To obtain an EEG dataset free of artifacts, the EEG epochs labeled as “artifact,” employing the epoch rejection criteria (EEGLAB, 2017), were removed from the EEG dataset (11 ± 5% of data has been on average removed).

The EEG segment associated with the period of “closed eye” (see Experimental Protocol) was used to detect the individual peak of the signal power spectrum within the traditional alpha frequency range (8 – 12 Hz), known as the individual alpha frequency (IAF), that is generally employed for the calculation of the individual EEG frequency bands, according to Klimesch (1999) as follows: theta [IAF – 6–IAF – 2], alpha [IAF – 2–IAF + 2], upper alpha [IAF –IAF + 2], beta [IAF + 2–IAF + 16], and gamma [IAF + 16–IAF + 30]. In the present study, alpha and upper alpha rhythms have been considered, being the most informative brain activity features related to motivational (Davidson et al., 1990; Harmon-Jones et al., 2010; Briesemeister et al., 2013) and attentive (Klimesch et al., 1998; Kelly et al., 2003; Sauseng et al., 2005) processes.

For both datasets, the global field power (GFP) was performed, representing a summary of the activity associated with the frontal EEG channels and to the two frequency bands of interest (alpha and upper alpha; Lehmann and Michel, 1990), according to previous studies (Ohme et al., 2009, 2010; Vecchiato et al., 2010, 2011) that employed the GFP as a reference-independent descriptor of the field potential (Skrandies, 1990).

The GFP formula is specified in the following equation:


(1) $$\begin{array}{c} GFP_{\alpha ,Frontal}=\;\frac{1}{N}\sum_{i=1}^{N}x_{\vartheta_{i}}{\left(t\right)}^{2} \end{array}$$

α represents the specific EEG band, Frontal represents the cortical area, N represents the number of electrodes related to a specific area, i represents the electrodes' index and x is the specific EEG sample at a time (t), filtered within the related EEG band (i.e., α) and for the specific channel i.

Afterward, the average GFP value on all the GFP values estimated over 1 s of EEG signal was calculated.

The approach/withdrawal (AW) index has been defined according to previous studies (Harmon-Jones et al., 2010; Briesemeister et al., 2013) and calculated employing the following formula:


(2) $$\begin{array}{r} \text{Approach withdrawal index}=\text{GFP}\alpha \_\text{right}-\text{GFP}\alpha \_\text{left} \end{array}$$

In particular, GFPα_right is associated to right electrodes (Fp2, AF4, AF6, and AF8) for the alpha (α) band, while GFPα_right is associated to left electrodes (Fp1, AF3, AF5, and AF7) for the alpha (α), generating an approach/withdrawal (AW) index where higher AW values indicate higher approach motivation and lower AW values indicates withdrawal motivation.

In addition, in this study, we have explored also the average values related to GFPupperα, related to all the frontal electrodes (Fp2, AF4, AF6, AF8, AF7, AF3, Fp1, AF5) for the upper alpha band, that we defined as “Frontal Upper Alpha.”

Finally, the neurometric of AW and “Frontal Upper Alpha” have been normalized for each second by the use of the mean and the standard deviation of the same neurometric calculated on the individual baseline (see “Experimental Protocol”) as shown in the following formula:


(3) $$\begin{array}{r} normalized\;index=\frac{index-{mean}_{baseline}}{{standard\;deviation}_{baseline}} \end{array}$$

### Autonomic Recording and Signal Processing

The Shimmer System (Shimmer Sensing, Ireland), with a sampling rate of 64 Hz, was employed for the recording of the Blood Volume Pulse (BVP) and GSR, respectively through the placement of a pulse oximeter on the thumb and of two electrodes on the second and third fingers of the non-dominant hand, according to published procedures (Measures et al., 2012).

The Pan-Tompkins algorithm has been used to obtain the HR signal from the BVP (Pan and Tompkins, 1985), while the LEDAlab software has been used for estimating the tonic component of the skin conductance (Skin Conductance Level, SCL; Benedek and Kaernbach, 2010), that was recorded with a constant voltage method (0.5 V).

The Emotional Index was performed employing and matching both SCL and HR signals, taking into account the circumplex model of affect plan (Russell and Barrett, 1999; Posner et al., 2005), where the coordinates of a point in space are defined respectively by the HR (horizontal axis) to describe the valence and by the SCL (vertical axis) to describe the arousal phenomena (Mauss and Robinson, 2009).

The Emotional Index represents a monodimensional variable, associated with the emotional state of a subject (Vecchiato et al., 2014b) and is defined by the following formula:


(4) $$\begin{array}{r} EI=1-\;\frac{\beta }{\pi }, \end{array}$$

where,


(5) $$\beta =\left\{ \begin{array}{l} \frac{3}{2}\pi +\pi -\vartheta \text{ if }GSR_{z}\geq 0,\;HR_{z}\leq 0,\\ \frac{\pi }{2}-\;\vartheta \;otherwise. \end{array}\right.$$

HR_z_ and GSR_z_ respectively represent the *Z*-score variables of HR and GSR, while ϑ is in radians and it is calculated as arctan (HR_z_, GSR_z_). The angle β varies between [−1, 1], as well as the values of the EI [−1, 1]. According to the result interpretation (Vecchiato et al., 2014b), negative (HR_z_ <0) and positive (HR_z_ > 0) values of the EI respectively indicate negative and positive emotions (valence).

### Eye-Tracking Recording and Signal Processing

The Tobii Pro X2-30 screen-based eye tracker (Danderyd, Sweden), with a sampling rate of 30 Hz, was employed for the recording of eye-tracking data. The eye tracker was placed within the Tobii Pro Mobile Device Stand (Tobii, 2019), a solution designed for mobile device testing, and then plugged into the acquisition computer where the software Tobii Studio 3.4.8 (Danderyd, Sweden) was running and performing the eye-tracking data acquisition. In addition, the Epiphan's DVI2USB 3.0 (Palo Alto, CA, United States), a high-performance USB video grabber for lossless video capture, was employed to mirror the user screen related to the Samsung Galaxy S8 smartphone to Tobii Studio 3.4.8 with a resolution of 1,920 × 1,200 at 60 fps, to achieve an accurate matching of the transferred images and eye-tracking data. Tobii Studio 3.4.8 has been used to draw the areas of interest (AOI) associated with the entire user screen and to the four advertisements, for the time segment related to each article reading. The same software was employed to extract fixation data associated with each AOI and for each article reading, using the Tobii I-VT fixation filter (Danderyd, Sweden) (Olsen, 2012). The visual attention metric was performed by dividing the total number of fixations elicited by an AOI (i.e., advertisement) for the total number of fixations recorded on the user screen AOI, concerning each article reading. In addition, the viewing time metric associated with each advertisement was obtained, known also as “visit duration” and defined as the interval of time in seconds between the first fixation on the AOI and the next fixation outside the AOI (Tobii, 2016).

### Self-Reports

After the exposure to each article, subjects have been asked to fill up a short questionnaire featured by a 7 points Likert scale. In particular, they have been asked: “Thinking about the article you just read how much you consider it:”

- Pleasant
- Annoying
- Having an advertising intent

The questionnaire variables associated with the self-reported measures of pleasantness, annoyance, and advertising intent have been normalized between 0 and 1 and will be reported in percentage.

### Performed Analysis

As described in Section “Experimental Protocol,” two groups (“X” and “Y”) have been generated to show the same four articles to all participants, where only the advertising format associated with each article was swapped across groups. Considering that each participant saw both display and native advertising formats, paired Sample *t*-Test was performed to compare:

- Regarding eye tracking metrics, the visual attention/viewing time generated by all display ads included in the article (averaged for all articles containing display ads) vs. the visual attention/viewing time generated by all native ads included in the article (averaged for all articles containing native ads).
- Regarding neurometrics, the level of the approaching motivation (AW)/EI elicited by an article containing display ads (averaged for all articles containing display ads) vs. the level of approaching motivation (AW)/EI elicited by an article containing native ads (averaged for all articles containing native ads).
- Regarding self-report, the degree of pleasantness/annoyance/advertising intent associated with an article containing display ads (averaged for all articles containing display ads) vs. the degree of pleasantness/annoyance/advertising intent associated with an article containing native ads (averaged for all articles containing native ads).

## Results

### Visual Attention

**Figure 5:**
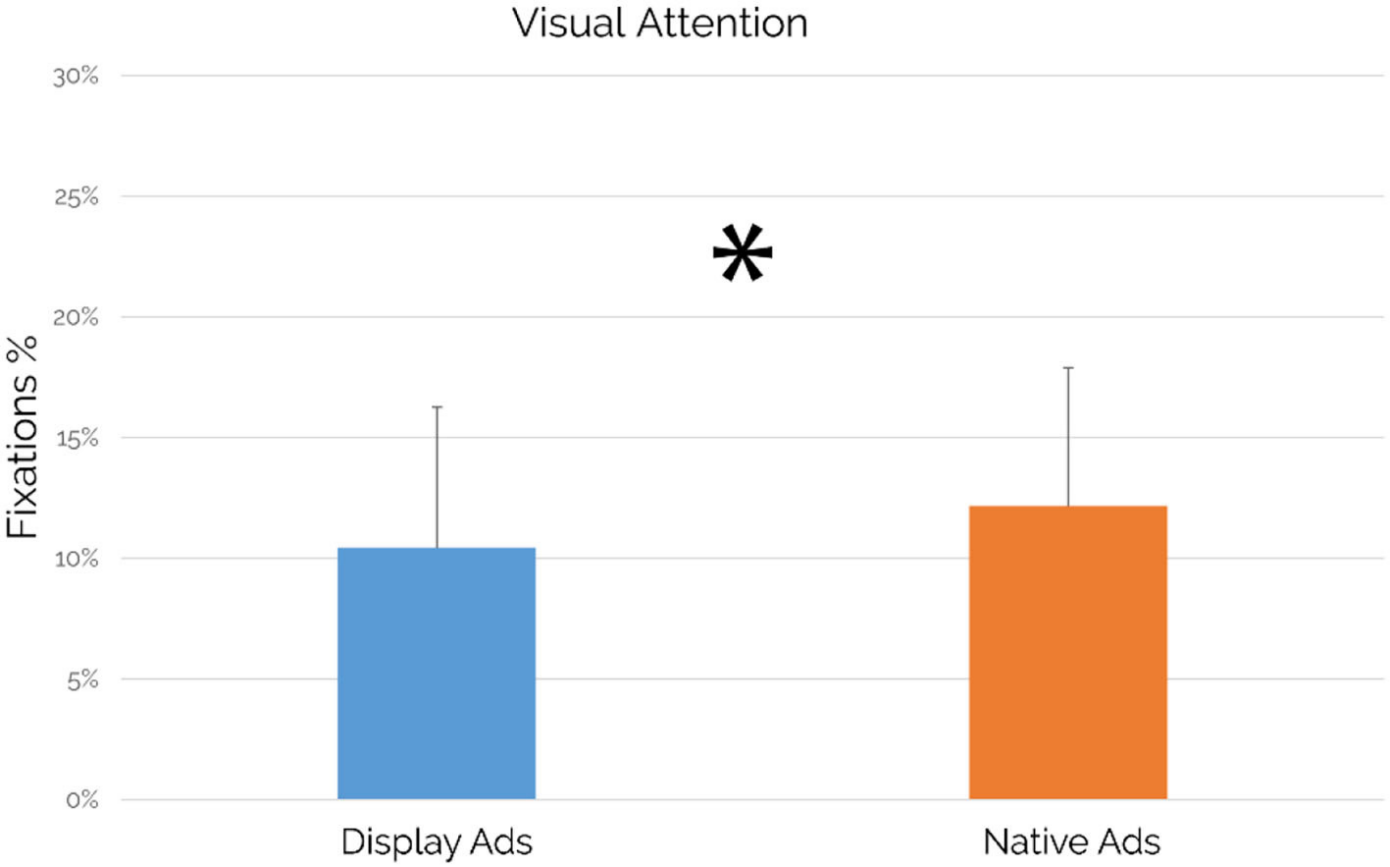
Visual attention: comparison between the display and native ads (*n* = 49). Native ads showed a statistically significant increase in visual attention values in comparison to display ads (*p* = 0.021, *d* = 0.35). Error bars represent SD.

Regarding the comparison performed between the visual attention generated by all display ads included in the article (averaged for all articles containing display ads) and the visual attention generated by all native ads included in the article (averaged for all articles containing native ads), results showed a statistically significant increase of visual attention for native ads in comparison to display ads (*p* = 0.021; see Figure 5).

### Viewing Time

**Figure 6:**
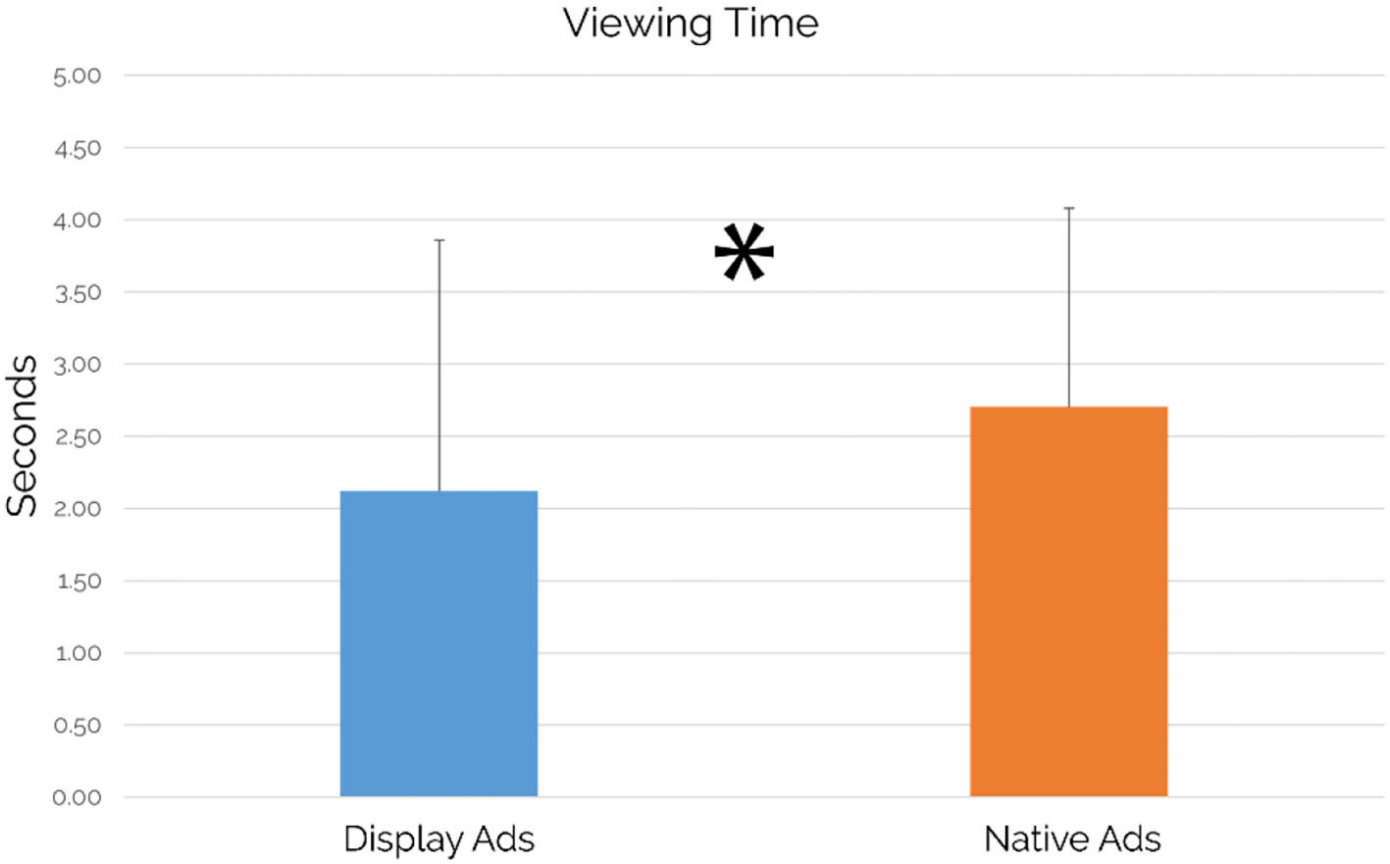
Viewing time: comparison between the display and native ads (*n* = 49). Native ads showed a statistically significant increase in Viewing time values in comparison to display ads (*p* = 0.041, *d* = 0.31). Error bars represent standard deviation.

Regarding the comparison performed between the viewing time elicited by all display ads included in the article (averaged for all articles containing display ads) and the viewing time elicited by all native ads included in the article (averaged for all articles containing native ads), results showed a statistically significant increase of viewing time for native ads in comparison to display ads *(p* = 0.041; see Figure 6).

### Approach/Withdrawal

Regarding the comparison performed between the level of the approaching motivation (AW) associated with an article containing display ads (averaged for all articles containing display ads) and the level of the approaching motivation (AW) associated with an article containing display ads (averaged for all articles containing display ads), results showed no significant differences (*p* = 0.408).

### Frontal Upper Alpha Power

**Figure 7:**
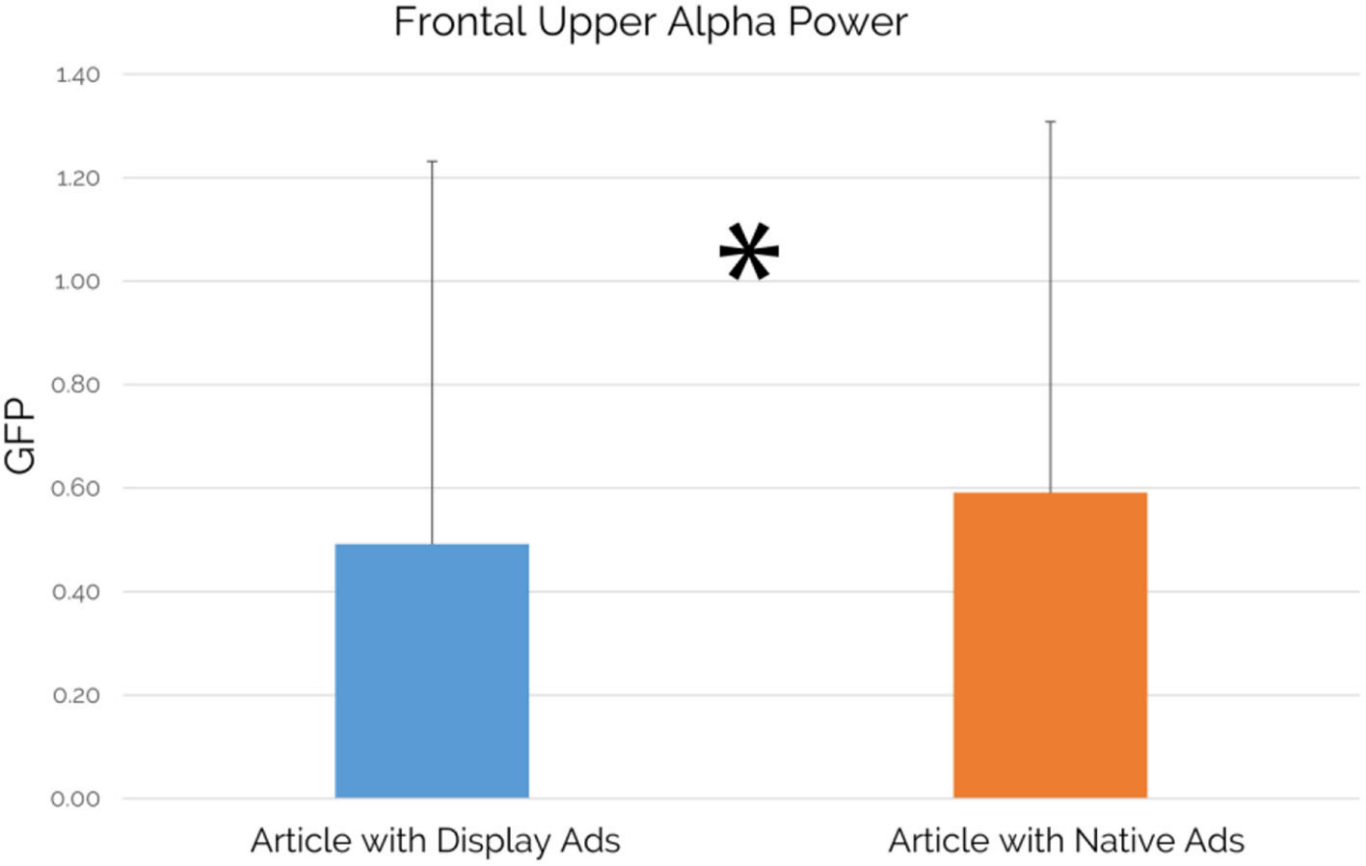
Frontal Upper Alpha Power: comparison between an article containing display ads and an article containing native ads (*n* = 49). An article containing native ads showed a statistically significant increase in Frontal Upper Alpha Power values in comparison to an article containing display ads (*p* = 0.047, *d* = 0.3). Error bars represent standard deviation.

A further investigation has been performed for the alpha power while comparing between the level of the Frontal Upper Alpha Power associated with an article containing display ads (averaged for all articles containing display ads) and the level of the Frontal Upper Alpha Power associated with an article containing native ads (averaged for all articles containing native ads) has been performed. Results showed a statistically significant increase of Frontal Upper Alpha Power for an article containing native ads in comparison to an article containing display ads (*p* = 0.047; see Figure 7).

### Emotional Index

Regarding the comparison performed between the level of the EI associated with an article containing display ads (averaged for all articles containing display ads) and the level of the EI associated with an article containing native ads (averaged for all articles containing native ads), results showed no significant differences (*p* = 0.401).

### Self-Reported Pleasantness

Concerning the comparison performed between the level of the self-reported Pleasantness associated with an article containing display ads (averaged for all articles containing display ads) and the level of the self-reported Pleasantness associated with an article containing native ads (averaged for all articles containing native ads), results showed no significant differences (*p* = 0.096).

### Self-Reported Annoyance

Regarding the comparison performed between the level of the self-reported Annoyance associated with an article containing display ads (averaged for all articles containing display ads) and the level of the self-reported Annoyance associated with an article containing native ads (averaged for all articles containing native ads), results showed no significant differences (*p* = 0.907).

### Self-Reported Advertising Intent

**Figure 8:**
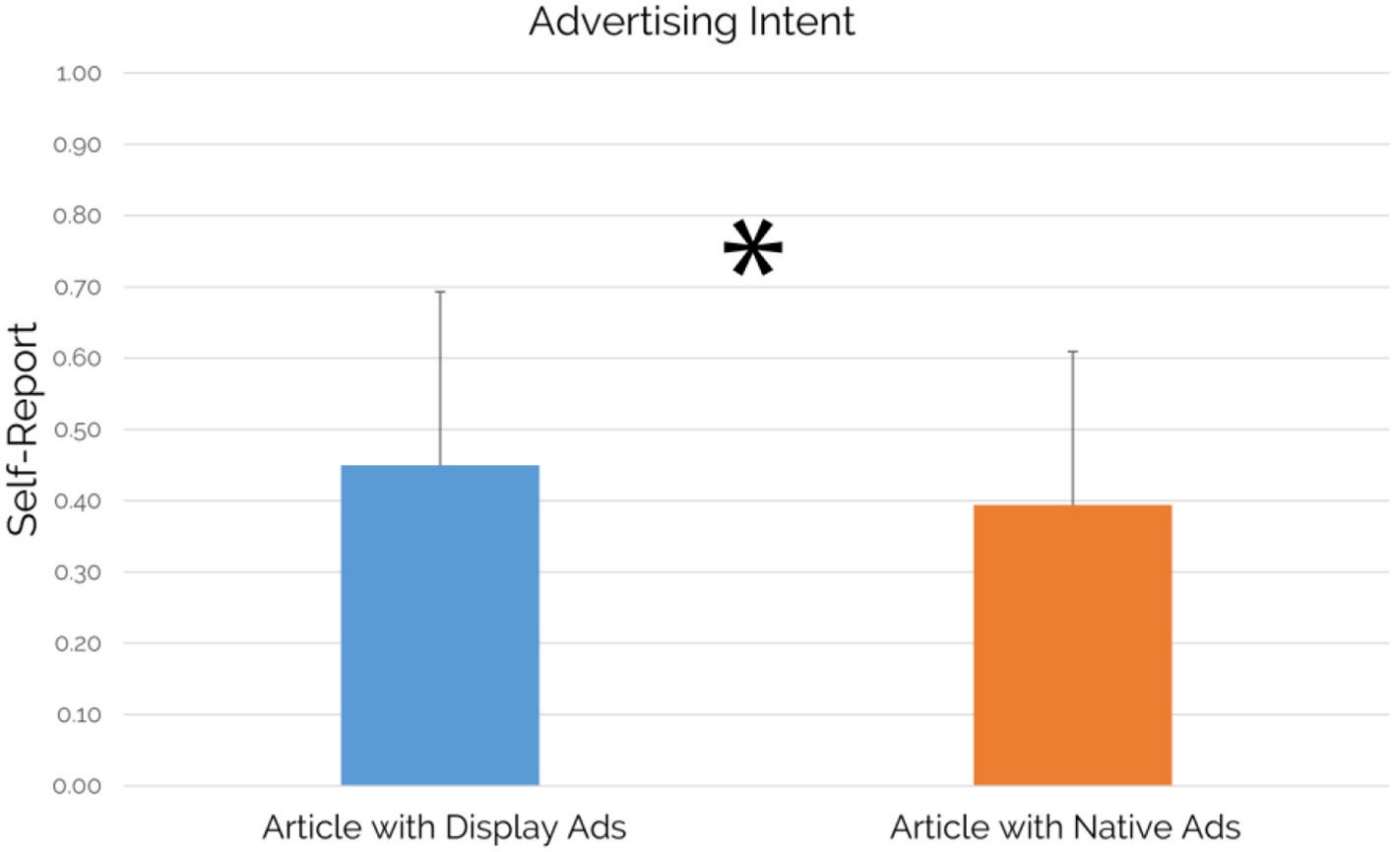
Self-reported Advertising Intent: comparison between an article containing display ads and an article containing native ads (*n* = 49). An article containing display ads showed a statistically significant increase in self-reported Advertising Intent values in comparison to an article containing native ads (*p* = 0.035, *d* = 0.31). Error bars represent standard deviation.

Concerning the comparison between the level of the self-reported Advertising Intent associated with an article containing display ads (averaged for all articles containing display ads) and the level of the self-reported Advertising Intent associated with an article containing native ads (averaged for all articles containing native ads), results showed a statistically significant increase of the self-reported Advertising Intent for an article containing display ads in comparison to an article containing native ads (*p* = 0.035; see Figure 8).

## Discussion

During the experiment, the subjects have been exposed to articles including native ads or display ads while their neurophysiological responses were collected, to investigate, during the experience, affective and cognitive processes elicited by the target stimuli. Beyond the main findings related to the case study, which will be extensively discussed in the next paragraphs, we believe that the effectiveness of the integrated set of tools that have been employed to investigate the digital stimuli of interest would be the most deserving of attention in the first instance. In a context where advertising research still relies mostly on traditional research methods such as questionnaires, focus groups, and interviews along with web metrics (e.g., impressions) to assess advertising efficacy, our integrated approach demonstrated how to address some of the weaknesses that the advertising industry is dealing with. First, the investigation of the users' gaze behavior by the eye-tracking technique provides an objective measure of what is seen, in comparison to the results that are possible to obtain by web advertising platforms, this latter providing metrics such as the impression metric, that doesn't reliably reveal if an ad is seen. In particular, our research protocol, by the use of the “viewing time” and “visual attention” metric, showed how to map the attention of multiple users to a stimulus of interest, in terms of time and distribution, providing clear answers to the following questions: “How long, in seconds, do we focus on a specific ad?” “Concerning the attention elicited by the entire page, how much attention a specific ad is capable to draw?” The adoption of the mobile device stand along with the X2-30 fixed eye tracker allowed us to accurately track the user's gaze on the employed smartphone, without requiring subjects to wear eye-tracker glasses or adding further constraints. A the same time, an integrated set of tools that also allows the recording of the EEG, BVP, and GSR provides additional information that cannot be obtained by traditional research tools in a similar context. Such recordings provide insights related to the level of the approaching motivation and EI elicited during the user experience, offering the experts the following advantages during the investigation of affective and cognitive processes:

- Data samples are collected during the experience, without the risk of distracting subjects from their task, and not only in the following phases as usually performed in advertising research protocols that include only traditional methods.
- It is possible to assess sub-phases of each experience, through the segmentation of neurophysiological data (e.g., segmentation of the acquired signal related to articles showing native or display ads)
- Considering that the psychological processes elicited by digital ads might last only a few seconds, it is important to highlight that the techniques used make it possible to explore such processes on a scale from milliseconds to seconds.
- Technologies' portability and non-invasiveness
- The opportunity to study phenomena that can occur below the awareness' threshold, hardly accessible by individuals, but that still plays a key role in driving users' behavior in digital contexts.
- Integration with traditional methods (e.g., self-reports related to the perception of articles containing display/native ads)

Last, but non-least for importance in this context, it is worth highlighting that the digital stimuli explored by the aforementioned integrated approach have been accurately prototyped in high fidelity, to control the noise that usually negatively affects the proper investigation of digital stimuli. In other words, high fidelity prototyping allows researchers to deal with the continuous variability of digital ads, over which no type of control is possible in normal conditions, turning an extremely challenging operation into a valuable opportunity. This feature played a key role in the adopted protocol, where all subjects have been exposed to the same version of each article except for the advertising format (native/display) that was swapped across groups, allowing us to compare the impact of native and display advertisements while controlling all the other article features.

Focusing on the case study's main findings, our results confirmed the success of native advertising both in terms of visual attention and viewing time while comparing it with the traditional display advertising format. The “visual attention” metric, based on fixations, refers to the distribution of the visual attention on the entire article page, allowing the detection of the attention elicited by specific AOIs, represented by the advertisements. In particular, the native ads available in the article were able to draw altogether the 12% of the visual attention elicited by the whole article, significantly higher than the 10% of visual attention elicited by the display ads (see Figure 5). Results related to the viewing time metric, known also as “visit duration” and defined as the interval of time in seconds between the first fixation on the AOI and the next fixation outside the AOI (Tobii, 2016) showed that native ads were capable to grab the attention of the users for a significantly longer duration and with an increase of 584 ms in comparison to display ads, as shown by the results associated with the viewing time metric (see Figure 6). At the same time, it is important to point out that the observed differences are featured by small effect size. Beyond the eye-tracking results aimed to provide further insights associated with the user attention, another important area of investigation was related to the perception of articles containing display or native ads in terms of approaching motivation, EI, pleasantness, annoyance, and advertising intent. Regarding the concerns raised from some authors, mainly related to counter-current results highlighting a more positive evaluation of display ads vs. native ads (Harms et al., 2019) and to the deceptive features of native ads as a source of negative effects (Fransen et al., 2015; Wojdynski, 2016a; Taylor, 2017; Han et al., 2018; Iversen and Knudsen, 2019), our study aimed to integrate neurophysiological and traditional measures to further investigate user perceptions associated to articles containing display or native ads to provide further understanding. In this context, our results showed that no significant differences between an article containing display ads and an article containing native ads in terms of approaching motivation, EI, pleasantness, and annoyance have been detected. The approaching motivation, based on the approach/withdrawal (AW) index, was not affected by the advertising format. In other words, users are similarly motivated to engage/disengage with articles containing native and/or display ads. Similarly, the EI, investigated through the Emotional Index (Vecchiato et al., 2014b), is not influenced by the ad format itself. Despite that, considering the crucial role of emotion in terms of advertising effectiveness, as already mentioned by Zajonc 40 years ago (Zajonc, 1980), future research could further explore the brain response to different ad formats in deeper brain regions, by the use of neuroscientific tools featured by higher spatial resolution than those employed in this study. Consistently with the results obtained on the neurophysiological measures of approaching motivation and EI, the self-reported measures of pleasantness and annoyance did not show a clear preference for one of the two advertising formats. On the other hand, focusing on the users' ability to detect the advertising intent of an advertisement, the article containing display ads has been perceived with a significantly higher advertising intent in comparison to an article containing native ads, even if the observed difference was featured by small effect size. Taking into account all the results obtained through the investigated measures, the following consideration arises: even if display ads are seen with a lower extent than native ads and users self-reported higher advertising intent for an article containing display ads, our results demonstrated that there is no clear users' preference for one of the two advertising format, contributing to provide further evidence to the current debate on the preference associated with one of the two advertising formats and on the negative evaluation of native ads due to their deceptive features. At the same time, the capability of native ads to draw significantly higher attention than display ads should be taken into account by advertisers. In this direction, display ads are negatively affected by the user's tendency to ignore banner-like information or anything else that resembles an advertisement as demonstrated by previous research studies (Nielsen, 2007; Pernice, 2018). In this context, our study showed that the display format led to a higher recognition of the advertising intent that affects its power in drawing attention, highlighting the increasing abilities of users to recognize such advertising format along with the development of a set of strategies to resist to it (Fransen et al., 2015). An opportunity that we missed in this study, but that we encourage future research to focus on, is related to the assessment of the impact that the reported differences could have played on the participants' behavior associated with the use of the selected products. For instance, after the exposure to the web pages showing the advertisements, participants could be asked to use a camera or sunglasses (seen in ads during the experiment) for a given time, aiming to investigate how the different ads, affect the behavior associated with the use of the selected objects. Interestingly, our study pointed out a different activation of the pre-frontal cortex in terms of upper alpha rhythms during the experience, with higher values when reading an article including native ads in comparison to the reading of articles including display ads, although such difference was featured by small effect size. Previous neurophysiological research widely investigated brain cortical behavior concerning the different components of activation and pointed out the key role of alpha rhythms in this regard (Klimesch et al., 1998; Aftanas and Golocheikine, 2001). In general, alpha activity has been recognized to have an inhibition effect over brain cognitive functions, so that the decreasing of alpha activity has been often correlated with the beginning of attentive processes (Jensen and Mazaheri, 2010; Händel et al., 2011; Uusberg et al., 2013). However, attention is also a very wide and complex concept, for such a reason neuroscientific research shed light on the role of alpha sub-rhythms concerning different aspects of attention. In particular, Klimesch and colleagues (Klimesch et al., 1998) demonstrated that alpha desynchronization does not happen at the same time for external stimuli mental processing: while low alpha components are sensitive to pre-stimulus alertness, higher alpha component (i.e., upper alpha) reacts only once the stimulus is processed. Even more significant is the recent work of Milton and colleagues (Milton and Pleydell-Pearce, 2016), where it has been demonstrated that frontal upper alpha shows synchronizing behavior in the period between a stimulus cue and the actual stimulus presentation. In other words, upper alpha synchronization has been correlated with induced alertness and expectation phenomena. In this light, the results of the present study look coherent and consistent with previous scientific evidence, since higher upper alpha synchronization has been detected in correspondence of native ads, as a sign of induced expectation in the readers. Eye-tracking data actually confirmed that display ads, being affected by banner blindness, obtained lower visual attention than native ads. Focusing on such evidences, something that is less seen, reasonably could induce less expectations and even if this discussion has to be considered as pure deduction at this stage, it might really be worth exploring further in future studies. Finally, it is important to mention that the observed differences, as demonstrated by the analysis conducted on the effect size, were featured by a small effect. In this direction, we believe that there is still a chance that the effect's limited size might be due to the sample size, which influences the standard deviation of the results and it could be assessed by replicating the study on a larger experimental sample as suggested by a recent study (Vozzi et al., 2021).

## Conclusion

The present study, through an innovative research protocol specifically designed for advertising research on smartphone devices, reported interesting findings associated with the perception of native and display ads. The investigation of user perceptions has been conducted by the integration of both neuroscientific and traditional techniques, with the aim to deeper explore psychological processes that play a key role in advertising research. According to our objectives, our study demonstrated the effectiveness of an integrated set of tools, shedding more light on the perception of two of the most widely used advertising formats in terms of attention, approaching motivation, EI, pleasantness, annoyance, and advertising intent. Native ads are generally harder to be recognized by the user as advertisements because they match the look, feel, and function of the media format in which they appear. In addition, the only cue that helps the user to identify them is represented by hidden words such as “branded” or “sponsored,” often shown in small size. Such deceptive feature of native ads looks to affect the visual attention and the viewing time elicited by an advertisement on smartphone devices, as shown by the results obtained through the comparison of display ads and native ads on those two indices of interest. In particular, the higher visual attention and viewing time elicited by native advertisements in comparison to traditional display advertisements indicates the effectiveness of this more recent type of advertisement format. In this direction, the investigation of eye-tracking data represents a great opportunity for advertisers and associations operating in the field of digital advertising (e.g., IAB) to develop more precise measurements (e.g., mobile viewable impression) based on objective data. At the same time, the analysis of neurometrics allows to objectively measure affective and cognitive processes elicited by articles containing different advertising formats, filling the current gap and well-integrating the results obtained through the traditional self-reports that mainly rely on how users describe advertisements. The integrated approach showed no clear preferences associated with articles containing display or articles containing native ads, although users reported a higher advertised intent for articles containing display ads. Our study did not report therefore evidence supporting the idea that native ads, because of their deceptive features, could negatively affect the perception of users to a greater extent than display ads.

The results associated with visual attention (eye-tracking) and the GFP in upper alpha (EEG), this latter showing higher upper alpha synchronization in correspondence of native ads, could support the idea that something that is less seen, reasonably could induce fewer expectations. The mentioned deduction, as previously mentioned, would require more studies to be confirmed. For example, the same tasks (i.e., an article to read) should be repeated against a control condition where no ads should be included, to verify if this expectation-related upper-alpha synchronization is still lower than in the case of articles including native ads. Also, it would be interesting to verify if a consequent desynchronization happens while the user clicks on the native ad and the redirection toward the advertising landing page is triggered. Unfortunately, these implications were not expected while designing the experiment, however, we will take these results into serious consideration for future studies as well as encourage further research in this way. Another aspect that could be interesting to explore in future studies, through the use of neurophysiological measures, is related to the comparison of several solutions of native ads based on the manipulation of the level of their deceptive features. In addition, in recent years we have witnessed an exponential growth of neuromarketing online solutions, based on webcam eye-tracking and facial coding, aimed to provide a better understanding of mechanisms underlying the visual attention and emotional response elicited by digital marketing stimuli. Despite most of such solutions are not still mature and would require additional testing, the promising technological advances in this direction will certainly allow in the next future to explore neurophysiological measures with a more flexible approach, reducing costs and analysis times and at the same time preserving data accuracy. Future research could play a key role in this context, where such a powerful set of online technologies need further exploration and validation.

## Data Availability Statement

The datasets presented in this article are not readily available because release of study data will require approval of local ethics authority. Requests to access the datasets should be directed to FB (fabio.babiloni@uniroma1.it).

## Ethics Statement

The studies involving human participants were reviewed and approved by Sapienza University of Rome Ethical Committee in charge for the Department of Molecular Medicine. The patients/participants provided their written informed consent to participate in this study.

## Author Contributions

EM and MM: conceptualization. AS, CS, and EM: formal analysis. PC and AS: investigation. GC and GB: methodology. MM: project administration. AM, AV, and VR: resources. GF, AV, and GB: software. MM, AT, and FB: supervision. MM, AS, and CS: writing—original draft. MM, PC, GF, and GC: writing—review and editing. All authors contributed to the article and approved the submitted version.

## Funding

This research was co-funded by European Commission through the Horizon2020 projects WORKINGAGE: Smart Working environments for all Ages (GA n. 826232), SAFEMODE: Strengthening synergies between Aviation and maritime in the area of human Factors toward achieving more Efficient and resilient MODE of transportation (GA n. 814961), MINDTOOTH: Wearable device to decode human mind by neurometrics for a new concept of smart interaction with the surrounding environment (GA n. 950998), FITDRIVE: Monitoring devices for overall FITness of Drivers (GA n. 953432), and H2020-SESAR-2019-2 project ARTIMATION: Transparent artificial intelligence and automation to air traffic management systems (GA n. 894238).

## Conflict of Interest

MM, PC, GF, GC, AM, AS, CS, AT, GB, and FB were employed by BrainSigns, Rome, Italy. The remaining authors declare that the research was conducted in the absence of any commercial or financial relationships that could be construed as a potential conflict of interest.

## Publisher's Note

All claims expressed in this article are solely those of the authors and do not necessarily represent those of their affiliated organizations, or those of the publisher, the editors and the reviewers. Any product that may be evaluated in this article, or claim that may be made by its manufacturer, is not guaranteed or endorsed by the publisher.
